# TensorCSBP: A Tensor Center-Symmetric Feature Extractor for EEG Odor Detection

**DOI:** 10.3390/diagnostics16050789

**Published:** 2026-03-06

**Authors:** Irem Tasci, Ilknur Sercek, Yunus Talu, Prabal Datta Barua, Mehmet Baygin, Burak Tasci, Sengul Dogan, Turker Tuncer

**Affiliations:** 1 Department of Neurology, School of Medicine, Firat University, Elazig 23119, Turkey; 2 Department of Digital Forensics Engineering, College of Technology, Firat University, Elazig 23119, Turkey; 3 School of Business (Information System), University of Southern Queensland, Toowoomba 4350, Australia; 4 Department of Computer Engineering, College of Engineering, Erzurum Technical University, Erzurum 25050, Turkey; 5 Vocational School of Technical Sciences, Firat University, Elazig 23119, Turkey; btasci@firat.edu.tr

**Keywords:** TensorCSBP, EEG odor detection, EEG signal classification, explainable feature engineering, Directed Lobish

## Abstract

**Objective**: Accurate odor classification from EEG signals requires informative and interpretable features. Although Local Binary Pattern (LBP) and variants such as the center-symmetric binary pattern are widely used, they lack sufficient explainability and tensor-level implementations. Additionally, neuroscientific understanding of odor processing remains limited. **Methods**: We propose Tensor Center-Symmetric Binary Pattern (TensorCSBP), a novel tensor-based feature extractor designed for EEG odor analysis. TensorCSBP is integrated into an explainable feature engineering (XFE) pipeline with four steps: (1) TensorCSBP for feature generation, (2) CWNCA for feature selection, (3) tkNN classifier for decision making, and (4) DLob method for symbolic interpretability. **Results**: TensorCSBP XFE was evaluated on a newly collected 32-channel EEG dataset for odor detection. It achieved 96.68% accuracy under 10-fold cross-validation. **Conclusions**: The information entropy of the DLob symbol sequence was 3.5675, demonstrating the richness of the interpretability output. Significance: This study presents a high-accuracy, explainable, and computationally efficient model for EEG-based odor classification. TensorCSBP bridges low-level signal patterns with symbolic neuroscience insights, offering real-time potential for BCI and clinical applications.

## 1. Introduction

The sense of smell (olfaction) is one of the basic senses that directly affects quality of life and plays a decisive role in human behavior, memory formation, emotional responses, and decision-making processes [1,2]. The olfactory system differs from other sensory systems because it has direct connections with the limbic system and does not rely on an initial thalamic relay in the same way as other modalities [3]. The body processes smells through neurophysiological mechanisms that link basic sensory functions to emotional and cognitive responses [4,5]. The complex nature of these interactions requires scientists to study brain odor processing, which has triggered neuroscience and behavioral science researchers to conduct more studies in this field [6]. However, the temporal and spatial complexity of brain activity related to odor makes it difficult to analyze this process objectively [7,8].

Electroencephalography (EEG) is a high-resolution, non-invasive, portable, and cost-effective neurophysiological recording method that is widely used in the assessment of various mental states [9,10].

EEG technology allows scientists to track brain signals in real time during the fast-paced process of smelling odors [11]. Scientists can perform detailed studies of brain sensory responses through the various signal paths and shifting EEG signal patterns [12]. The extraction of meaningful features from EEG signals becomes challenging because olfactory perception-specific EEG responses show low amplitude levels that differ between individuals and get affected by external environmental elements [13]. Current studies often attempt to overcome these challenges using complex deep learning (DL) models [14,15]; however, the high computational cost and limited explainability of such models raise trust issues, particularly in clinical and neuroscientific applications [16].

In this paper, we have presented a new feature extraction method called Tensor Center-Symmetric Binary Pattern (TensorCSBP) that can classify odor perception from EEG signals with high accuracy and produce explainable results. TensorCSBP is a tensor-based center-symmetric feature extractor capable of processing multi-channel EEG data. The proposed system performs classification while simultaneously enabling neurophysiological interpretation through DLob-based symbolic explanations derived from regional brain activity patterns [17]. In this context, the primary objective of this study is to present a machine learning framework that is both highly accurate and interpretable in EEG-based odor perception classification. The proposed model operates at reduced computational expenses compared to standard deep learning systems because it implements feature engineering methods to study system behavior. The method produces vital data that supports neuroscientific research and provides a reliable system for medical and industrial applications.

### 1.1. Related Works

The classification of EEG signals and the modeling of sensory cognitive states have attracted considerable interest in both the machine learning and neuroscience communities in recent years [18,19]. Most of the work in this field focuses on cognitive states such as motor imagery, stress, attention, mental performance, or epilepsy [20]. EEG analyses specific to smell, however, remain quite limited. Additionally, many studies use deep learning-based approaches to classify EEG signals in order to achieve high accuracy rates [21]. However, since the explainability level of these methods is low, the neurophysiological interpretability of decision-making mechanisms is often overlooked [16]. In this context, a summary of recent studies on the classification of different odor recognition tasks using EEG signals is presented in Table 1.

The recent studies summarized in Table 1 show that, despite the high accuracy rates reported in the field of EEG-based odor recognition, consistency issues with the method persist. Using an SVM-based approach that supports classical methods, Hou et al. [22] successfully distinguished five different odor intensities and pleasant-unpleasant binary distinctions with an accuracy rate of 92–94%, demonstrating the effectiveness of their method. However, a significant decline in performance is observed as the number of classes increases.

Indeed, in Kato et al.’s [23] ten-class experiment, the accuracy rate remained only between 14% and 55%. Ouyang et al. [29] achieved 95% emotional state classification accuracy through their CBRNet model, which combined CNN and BiLSTM architectures, but they did not explain the biological processes that led to their model predictions. Naser and Aydemir [26] achieved a classification accuracy of 88% through kNN-based classification when they used Hilbert amplitude features in their study. However, their study was limited to only 10 participants, and cross-subject generalization was not evaluated.

The current research on EEG-based odor recognition shows promising results, but different studies lack sufficient comparison methods. The main reasons for this include small and unbalanced sample sets, non-standardized experimental protocols, lack of open data sharing, and model decisions that are not sufficiently supported by neurophysiological explanations. The high classification performance of deep learning-based methods creates difficulties for their implementation in real-time systems because these methods require expensive computational resources. The current research lacks enough XAI solutions, which creates a critical knowledge gap that impacts medical safety and user confidence levels. The TensorCSBP study solves these problems through its creation of new EEG odor data and its development of a feature engineering system that explains results while using minimal computational resources.

### 1.2. Literature Gaps

The detected literature gaps are as follows:

- Based on our literature review, there is a lack of EEG odor classification studies because collecting EEG odor signals is difficult.
- Most researchers have used deep learning (DL) models to ensure high classification performance. However, DL architectures have high computational complexity; therefore, training DL models is expensive.
- In EEG signal analysis, most studies have focused on classification performance. As a result, explainable artificial intelligence (XAI) has been overshadowed.

### 1.3. Motivation and Our Model

Our main motivation in this research is to investigate the feature-extraction ability of the recommended TensorCSBP on EEG signals. To enhance the visibility of this study and address the lack of EEG odor detection research, we collected a new EEG odor detection dataset. The research employed TensorCSBP to achieve both excellent classification results and understandable findings. The research aimed to solve the existing knowledge gaps that researchers had not yet studied.

To fill the first literature gap, we collected a new EEG odor detection dataset. This dataset contains two classes: (i) good odors and (ii) bad odors.

The authors solved the second literature gap by developing their feature-engineering model. The research used TensorCSBP as the single feature extraction method to evaluate its ability to extract features. The development of a machine-learning pipeline required the implementation of feature selection and classification methods and an XAI result generator.

To fill the third literature gap, XAI results were generated by deploying the Directed Lobish (DLob) [17] XAI result generator.

In this research, we recommended an explainable feature-engineering (XFE) architecture. The recommended XFE framework has four main phases:

- TensorCSBP-based feature extraction,
- Cumulative Weighted Neighborhood Component Analysis [31] (CWNCA)-based feature selection,
- Classification using the t algorithm-based k-nearest neighbors (tkNN) classifier [32],
- DLob-centric XAI result generation.

**Figure 1:**
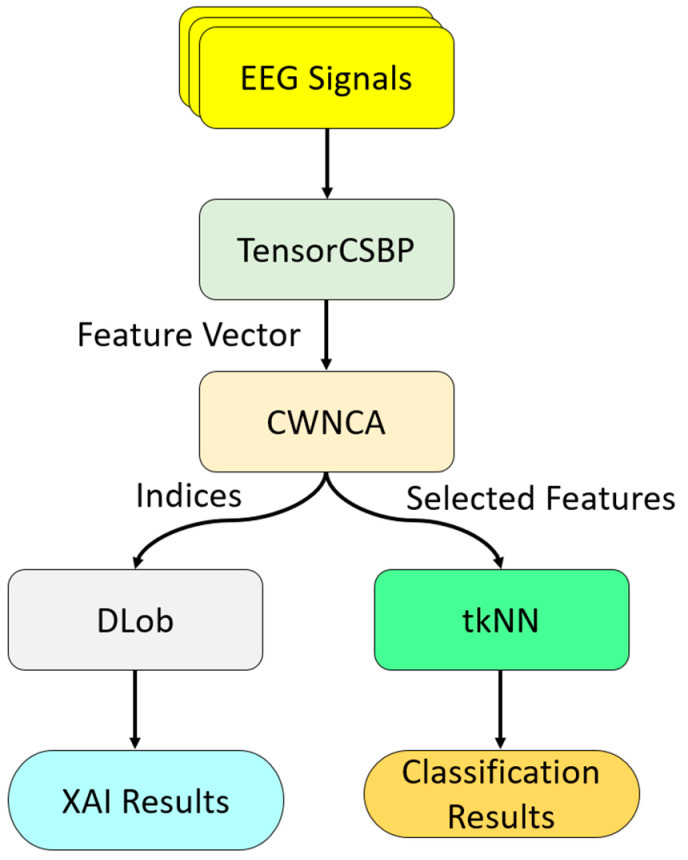
The general overview of the recommended TensorCSBP XFE model.

By utilizing these phases, a machine-learning pipeline was created. The features of the EEG signals were extracted using TensorCSBP. The salient features were selected using CWNCA [31]. The selected features and the indices of these selected features were used to obtain classification and XAI results, respectively. The selected features served as input to tkNN [32] for classification. The interpretable results were created using the indices of the selected features. By employing DLob [17], a cortical connectome diagram for odor detection was created. An overview of the presented model is shown in Figure 1.

## 2. Datasets

**Figure 2:**
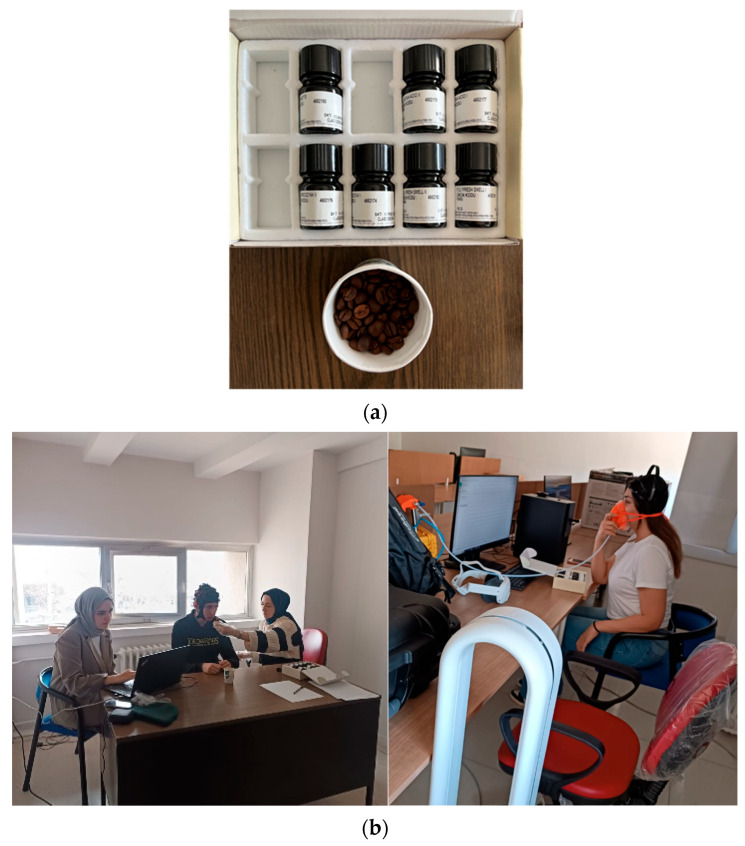
Dataset collection environment. (**a**) Odors; (**b**) EEG odor collection.

An innovative EEG odor classification dataset was collected in this research. The dataset was acquired using an Emotiv Flex brain cap, which has 32 channels and a sampling frequency of 256 Hz. Odor stimuli were grouped into two categories: (1) good and (2) bad odors. Odorants were selected to represent clearly distinguishable affective valence categories, consistent with established olfactory paradigms. Pleasant stimuli consisted of familiar everyday scents, whereas the unpleasant odor was deliberately prepared under controlled conditions to ensure consistent aversive perception. Stimuli were presented individually in a quiet, ventilated environment with standardized exposure intervals. Data were collected from 180 participants aged 18–49; 16 were female, and 164 were male. First, we presented the odors to the participants. Then, to minimize potential carryover effects between consecutive odor presentations, coffee was used as a neutral washout stimulus between consecutive odor presentations. Although coffee-scented beans are commonly employed as a “nasal palate cleanser,” current evidence does not demonstrate a significant advantage over alternative washout conditions, such as air or lemon, in odor identification tasks [33]. While experimental studies in animal models have reported long-term modulation of olfactory sensitivity following repeated exposure to coffee aroma [34], these findings do not pertain to immediate inter-trial effects. Therefore, coffee was employed as a conventional and standardized neutral stimulus rather than as a performance-enhancing intervention. The EEG signals were divided into 15-s segments. Accordingly, there are 571 good-labeled and 542 bad-labeled EEG segments, for a total of 1113 segments. The data collection process is shown in Figure 2.

### 2.1. The TensorCSBP XFE Model

To obtain classification and XAI results from the collected dataset, the TensorCSBP XFE was proposed. This machine-learning pipeline has four essential phases:

- TensorCSBP-based feature extraction;
- Feature selection with CWNCA;
- tkNN-centric classification;
- DLob-based XAI result generation.

**Figure 3:**
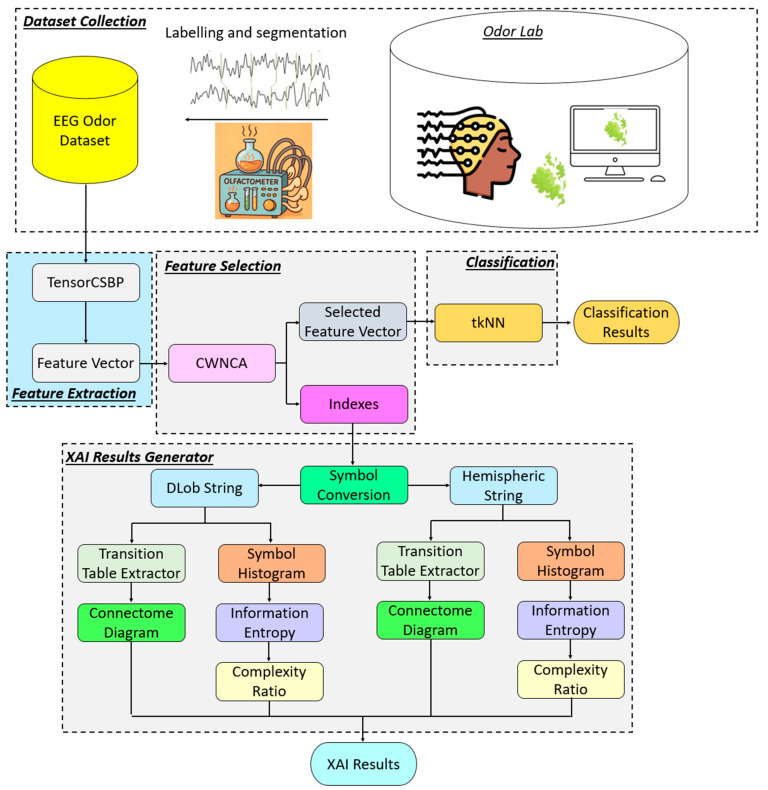
Graphical illustration of the proposed TensorCSBP XFE model.

The general block diagram of the introduced TensorCSBP XFE model is illustrated in Figure 3.

Figure 3 clearly shows that the presented XFE model generates features using TensorCSBP. The salient features were selected with the CWNCA [31] feature selector. The selected features were used as input to the tkNN [32] classifier to obtain the classification results. The indices of the chosen feature vector were used as input to DLob [17] to generate the XAI results. To provide details, the phases of the introduced TensorCSBP XFE are given below.

### 2.2. Feature Extraction

**Figure 4:**
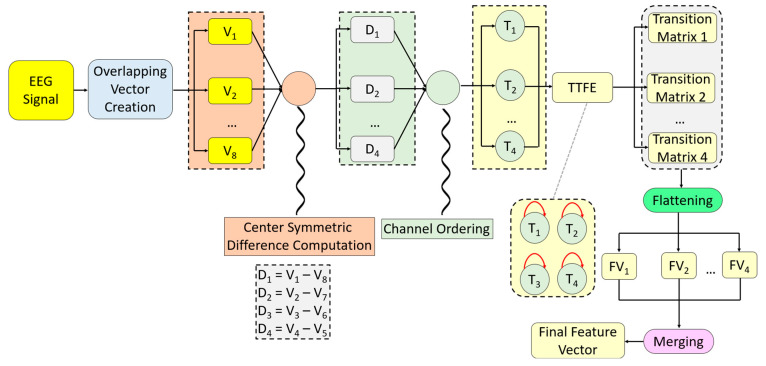
Graphical depiction of the TensorCSBP feature extractor. In this figure, the meanings of the abbreviations are given as follows. V: Vector, D: Distance; T: Transformed Signal; TTFE: Transition Table Feature Extractor; FV: Feature Vector.

In this section, the core innovation of the paper, TensorCSBP, is presented. The recommended TensorCSBP is a transformer-based feature extractor that combines CSTrans and TTFE. CSTrans produces four transformed signals from eight input vectors. These four signals are then given to the TTFE extractor, which yields four transition matrices. After the flattening and merging operations, the final feature vector is obtained. The steps of TensorCSBP and its graphical depiction are shown in Figure 4.

For clarity, the steps of the recommended TensorCSBP are given below.

*Step 1*: Create eight vectors by applying overlapping vector division.

(1) $$V_{j+1}=Signal\left(i+j,:\right),\;j\in \left\{0,1,\ldots ,7\right\},\;i\in \left\{0,1,\ldots ,L-7\right\}$$

Herein, V: vector, Signal: multichannel signal and L: length of the signal.

*Step 2*: Calculate center-symmetric differences of the eight vectors.

(2) $$D_{k}=V_{k}-V_{9-k},k\in \left\{1,2,\ldots ,4\right\}$$

where D: difference vector.

*Step 3*: Apply channel transformation to the difference signals to obtain transformed signals.

(3) $$T_{k}\left(ct:\;ct+N_{c}-1\right)=argsort\left(↓D_{k}\right),\;ct\in \left\{1,N_{c},\ldots ,N_{c}(L-8)+1\right\}$$

Here, T: transformed signal and $argsort\left(↓\right)$: sorting by descending.

*Step 4:* Repeat Steps 1–3 until the length of the EEG signal is reached.

*Step 5:* Generate four transition matrices using the transition table feature extractor.

(4) $$TM_{k}={\left[\begin{array}{ccc} 0 & \ldots & 0\\ \vdots & \ddots & \vdots \\ 0 & \ldots & 0 \end{array}\right]}_{N_{c}\times N_{c}}$$

(5) $$TM_{k}\left(T_{k}\left(a\right),T_{k}\left(a+1\right)\right)=TM_{k}\left(T_{k}\left(a\right),T_{k}\left(a+1\right)\right)+1,\;a\in \left\{1,2,\ldots ,L-1\right\}$$

Herein, TM: transition matrix and L: length of the transformed signal and four transition tables are created in this step.

*Step 6:* Flatten the feature matrices to obtain feature vectors.

(6) $$FV_{k}\left(q\right)=TM_{k}\left(w,u\right),\;w\in \left\{1,2,\ldots ,N_{C}\right\},u\in \left\{1,2,\ldots ,N_{C}\right\},\;q\in \left\{1,2,\ldots ,N_{C}^{2}\right\}$$

where FV: feature vector and the length of each feature vector is $N_{C}^{2}$.

*Step 7:* Merge the feature vectors to obtain the final feature vector.

(7) $$FF\left(q+N_{C}^{2}\left(k-1\right)\right)=FV_{k}\left(q\right)$$

Herein, FF: final feature vector with a length of $4N_{C}^{2}$.

These seven steps define the recommended TensorCSBP. Using the TensorCSBP feature-extraction method, features were extracted.

(8) $$X\left(s,:\right)=TCSBP\left(Signal_{s}\right),\;s\in \left\{1,2,\ldots ,N_{s}\right\}$$

where X: the created feature matrix, TCSBP(.): TensorCSBP and $N_{s}$: number of signals.

From a first-order perspective, the center-symmetric transformation is therefore a spatial contrast operator across EEG channels. Computing differences between symmetrically positioned channel vectors, it emphasizes relative hemispheric and regional activation patterns rather than absolute amplitudes. This allows the model to capture functional asymmetries that are commonly associated with sensory and affective cortical processing. The transition table feature extraction (TTFE) models the temporal evolution of these spatial dominance patterns. By quantifying transitions between ranked channel indices, it encodes dynamic changes in inter-regional relationships over time. Consequently, TensorCSBP represents EEG signals in terms of spatial contrasts and their temporal interactions, providing a structured and neurophysiologically interpretable feature space. These spatial–temporal patterns are subsequently evaluated by the feature selection and classification stages to determine whether consistent activation contrast structures differentiate odor categories. High classification accuracy indicates that the extracted contrast-transition patterns systematically vary between odor conditions. Therefore, the effectiveness of the proposed method is not derived from isolated channel amplitudes but from reproducible inter-regional activation relationships that distinguish odor classes.

### 2.3. Feature Selection

In the feature-selection phase, the CWNCA feature-selection function was used. In this phase, the selected features and their identities were obtained. CWNCA is an improved version of the NCA selector since it computes the optimal number of features using a cumulative-weight computation. The pseudocode of CWNCA is shown in Algorithm 1.
**Algorithm 1.** Pseudocode of the CWNCA feature selector.**Input:** Feature matrix (X) and threshold value (th) and real labels (y). **Output:** Selected feature matrix (SX) and the indices of the selected features ($in_{S}$). 01: **for** d = 1 to $N_{S}$ **do**//Applying min-max normalization to features. 02:   $X\left(d,:\right)=\frac{X\left(d,:\right)-\min\left(X\left(d,:\right)\right)}{\max\left(X\left(d,:\right)\right)-\min\left(X\left(d,:\right)\right)+\varepsilon }$ Herein, min(.): minimum value computation function, max(.): maximum value computation function and ε: epsilon. 03: **end for d** 04: wt=NCA(X,y);//Applying NCA where wt: weights, NCA(.): NCA feature selection function. 05: $in=argsort\left(↓wt\right)$;//Computation of the qualified indexes. Herein, in: the qualified feature indexes. 06: $of=CW\left(X,y,wt,in,th\right)$ where of: optimal number of the features, CW(.): cumulative weight computation. 07: **for** i = 1 to of **do** 08:   $SX\left(:,i\right)=X\left(:,in\left(i\right)\right);$ 09:   $in_{S}(i)=in(i)$; 10: **end for i**

Herein, the threshold value is selected as 0.9999.

### 2.4. Classification

The tkNN [32] classifier was used to generate classification results. The tkNN classifier is iterative and self-organizing. It produces both parametric and voted outcomes. By iteratively changing the parameters, parametric outcomes are generated. By applying iterative majority voting (IMV) [35], the voted outcomes are obtained. The pseudocode of the tkNN classifier is shown in Algorithm 2.
**Algorithm 2.** tkNN procedure.**Input:** Selected feature matrix (SX), bag of parameters (P) and actual labels (y). **Output:** Final outcome (${out}^{Final}$). 01: **for** i = 1 to $N_{P}$
**do** 02:   $out_{i}^{P}=kNN\left(SX,y,P_{i}\right)$//Parameter-based outcomes ($out^{P}$) generation. 03:   $acc\left(i\right)=\beta \left(out_{i}^{P},y\right)$//Classification accuracy (acc) computation step. Herein, β(.): classification accuracy computation function. 04:   $id_{acc}=argsort(↓acc)$//$id_{acc}$: the qualified identities. 05: **end for i** 06: **for** i = 3 to $N_{P}$
**do** 07:    $out_{i-2}^{V}=\omega \left(out_{id_{acc}(1)}^{P},out_{id_{acc}(2)}^{P},\ldots ,out_{id_{acc}(i)}^{P}\right)$//IMV applying. where $out^{V}$: voted outcome and ω(.): mode function. 08:   $acc\left(N_{P}+i-2\right)=\beta \left(out_{i-2}^{V},y\right)$//Classification accuracy computation. 09: **end for i** 10: $id_{max}=argmax\left(acc\right)$//Application of the greedy algorithm. 11: ${out}^{Final}=\left\{\begin{array}{c} out_{id_{max}}^{P},\;id_{max}\leq N_{P}\\ out_{id_{max}-N_{P}}^{V},\;id_{max}>N_{P} \end{array}\right.$

Algorithm 2 openly demonstrated that the tkNN classifier is a self-organized classifier.

### 2.5. XAI Results Generation

In this phase, the DLob method was used to create XAI results. DLob is a symbolic language, and each symbol corresponds to a specific brain area. This language contains 16 symbols, which are listed in Table 2.

According to the brain cap used, we employed *n* of these symbols (*n* ≤ 16). The second letters of these symbols indicate the hemisphere. We used the indices of the selected features as input to the DLob method to generate the XAI results. The procedure of the DLob method is demonstrated in Algorithm 3.
**Algorithm 3.** DLob XAI generator.**Input:** Indices of the selected features ($in_{S}$), look-up-table (LUT). **Output:** XAI results (XAI) 01: ct=1;//Counter definition 02: **for** i = 1 to $N_{S}$ **do** 03:   $Ch\left(ct\right)=\left[\left\lfloor \frac{in_{S}\left(i\right)-1}{N_{c}}\right\rfloor \left(mod\;N_{c}\right)\right]+1$//Channel (Ch) extraction. 04:   $Ch\left(ct+1\right)=\left[\left(in_{S}\left(i\right)-1\right)\left(mod\;N_{c}\right)\right]+1$ 05:   $S_{DLob}\left(ct\right)=LUT\left(Ch\left(ct\right)\right)$//$S_{DLob}$: DLob sequence. 06:   $S_{DLob}\left(ct+1\right)=LUT\left(Ch\left(ct+1\right)\right)$ 07:   ct+=2 08: **end for i** 09: Generate hemispheric sequence deploying the second letter of each generated DLob symbol. 10: Extract histogram of the DLob and hemispheric sequence. 11: Compute transition matrices to show connectome diagram. 12: Compute information entropy and information entropy-based complexity ratio of both sequences. 13: Present the generated sentences, histograms, transition tables and complexity ratios as XAI results.

## 3. Experimental Results

A new EEG odor dataset was collected, and a new XFE model, termed the TensorCSBP XFE architecture, was recommended. Using the TensorCSBP XFE architecture, classification and interpretable results were computed. The recommended model extracts features using TensorCSBP, selects salient features with CWNCA, computes classification results using the tkNN classifier, and generates interpretable results with the DLob XAI method.

The introduced XFE model is a parametric framework, and the utilized parameters are given below.

*TensorCSBP*

Number of vectors: 8;

Number of transformed signals/vectors: 4;

Length of the final feature vector: $4N_{C}^{2}$. In this research, we used a brain cap with 32 channels. Therefore, the length of the generated feature is computed as 4096.

*CWNCA*

Threshold value: 0.9999;

The number of selected features: 606.

*tkNN*

Parameters: k = [1, 2, …, 10], distances = [Euclidean, Spearman, City block, Cosine], weights = [Inverse, Squared Inverse, Equal];

Range of IMV: from 3 to 120;

Number of outcomes: 238;

Select the final outcome: Maximum classification.

*DLob*

Number of utilized DLob symbols: 14;

Number of utilized hemispheric symbols: 3;

Length of the sequences: 1212;

Utilized statistics: Histogram extraction, information entropy, complexity ratio, and transition table.

The introduced framework has linear time complexity because the utilized methods have linear time complexity. Unlike representative deep learning baselines, whose computational cost typically grows significantly with model depth and iterative backpropagation, TensorCSBP XFE operates with linear time complexity. This makes the proposed framework substantially more suitable for resource-constrained and real-time EEG odor applications where transparent decision logic is required. Therefore, we implemented the framework on a simple laptop with 32 GB of main memory, an Intel i9 processor, and Windows 11 Professional. MATLAB R2025a was used as the programming environment.

### 3.1. Classification Results

**Figure 5:**
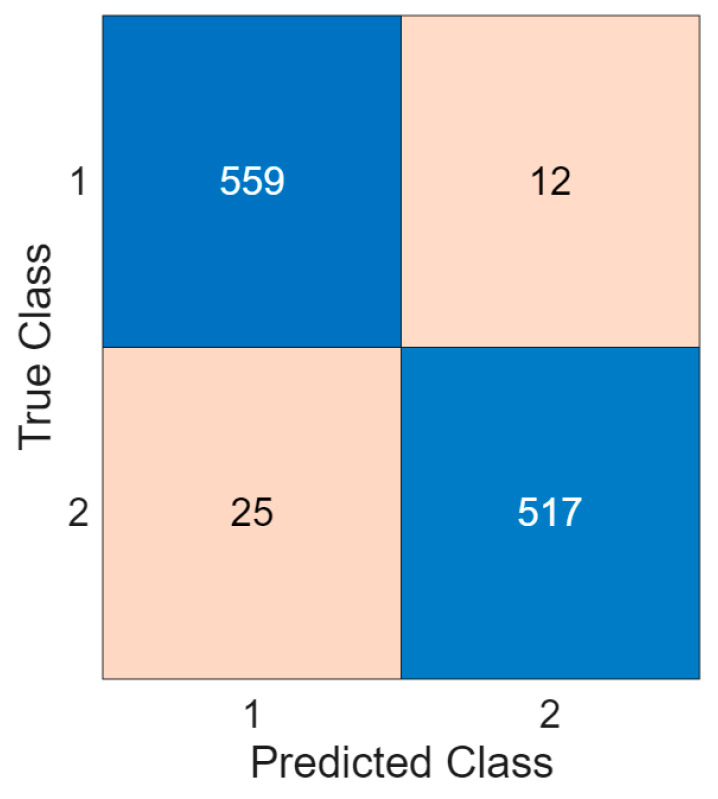
The computed confusion matrix. Herein, 1: good, 2: bad.

To evaluate classification performance, we used accuracy, sensitivity, specificity, precision, F1-score, and geometric mean. These metrics were computed from a confusion matrix, which is shown in Figure 5.

The computed classification performances are listed in Table 3.

Table 3 openly illustrated that the TensorCSBP framework attained over 95% results for all used performance metrics. Table 3 highlights that the recommended TensorCSBP framework yielded high classification performance.

**Figure 6:**
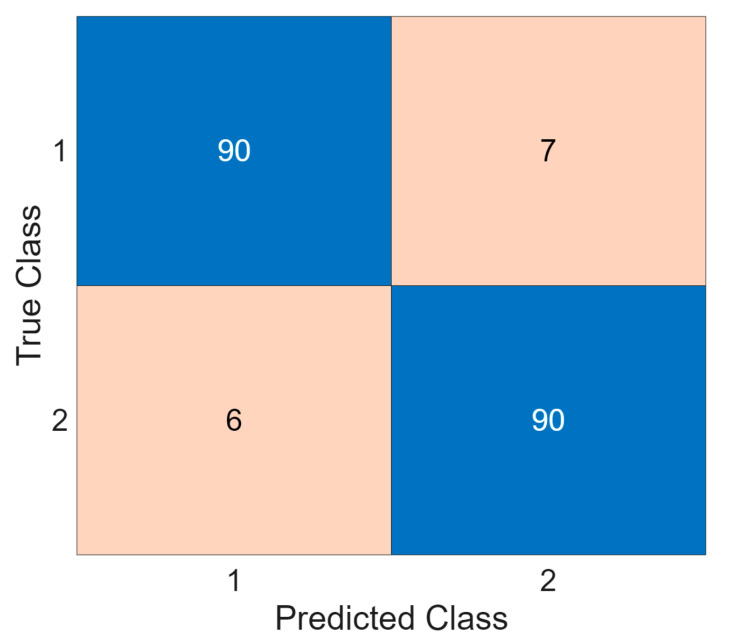
Confusion matrix from balanced male–female evaluation.

To further assess potential gender-dependent bias, a balanced subgroup evaluation was conducted using equal numbers of male (*n* = 16) and female (*n* = 16) participants. The resulting confusion matrix is presented in Figure 6. The model achieved an accuracy of 93.26%, with comparable class-wise sensitivity and specificity. The small performance deviation between genders indicates that the TensorCSBP XFE framework maintains stable decision boundaries across sexes despite the overall dataset imbalance. These findings suggest that the observed high performance is not driven by gender-specific bias.

### 3.2. Explainable Results

The second evaluation aspect is interpretability, which was assessed through the generated XAI outputs. To obtain XAI results, DLob and hemispheric symbol sequences have been generated. The generated sentences are given below.

DLob sentence:

OROzOLOROzPLPLOLOLPLTLPLFRFRPLFLFLCzORPRCzFRFRPRTLCLCzFRCLPLPRTRPRCRPzPRFRFROLOzFRFRPzFLPRORCLFLPLOLTROLFRFRFRTLCRPRCRTRFRFRPLTLTRCLFLFRFRTLPRPRORCzFLFLFLFzOzPzORPRPRTLPzPLPRPRPROzFRFLOLPRFLFLFRFROLPLPRCRFzFLOzORFLFRPzTLPRORPLOLFLFRCRPzPLOLFLFRFLCLFLTLFRFROROLPRFLFRFLPRPRCzFRTRFRCLFLPLOzCRFROzPLOzFRTLFLPRFLFLPzTLCLTLCLPRFLPRTLPRORPROLPRFLPLTLFLFLORPROzORTLPRFRCzPLPzTRPRTRFRFRFROzOLFRFLFLPLFRFROLOzTRPRFLFLCLTLFRCzPRPzFRFLFLFLFLFROLCRFRFLFLFLOROLFRFLOROLFLFLCLPLPLOLFRFLFzFLPRTLPRPRFLOzPRORCLCRPLCLPRPRFLOzFLPRPLOzFLFRFLFLTLPzPLOLPRPRFRFzOLTRFRTRFRFRTRCLPzPLPLOLOzOLFLFLTLFLTRFRFzTLOLCROLORFRFzFRFLCROLFRPRPLPLCLTLPLTLTRFRFRTRFLOROLPLPROzFRPROROLFLTLORPRPRPLOLPRPLOzTRFRORCLOLPzOLOzFRFLPLPLPRORFLFRPLOzFRFRFLPLFRFRCRPRPRTRFLFRFLPRFLCLOzOLPLFLCRFRPRPRPRTRPRPRFLFLOLPLFRCRCLCRTLCLOLORCzORPRCLFRFRCLFLTLFLOzPzPLORPROzFRFzPRFLCRTROzPRFLPRFRCzFLFzOLPLTRFRPROzPLOzCLCRFRFRFLOzOLPRPRTRCLTLTRPLOzPRFLCLPzFLPRPLFLPLCzFRFLCzORPLCRFRFRFLPLTROROzCLFLOLORFzFRPzPRPRPzPLPLFzFLFRPzTLPzOzPzPRCRPROLFRFRPROzFRFLPRCRCzCRPRCRPzTLPLFLTLPLFLCRPLOzTROLOzPLPzPRFzFRPROzFRFLPROLFRFRFRFRPLTRPLOLFLFRFRFRTLPRCRTRTLOLPRORTLOLFRFRFRTRPLOzFLFLTRFRCLTLOLFLCLTRORPLPLPRFLFLCzPRPzOzTLFLFRFLOLPRFRTROLPLFRFzFRFRPLOLPRPRCLPRCRPRORCzFLFLFzFRFRTRPLCzFLFRFRTRTRPRTRFRORPRCRPRTLOLTRCRPLFLCLFLFLCRPRPRFLPLCzOROLTRPRTLCRFRFRORFRCzFRFLOzOROzFLFzFLFLCzTLPLOzPLCzFLFLFRTRPRORPRTRORPLTRCLPLPROzPzOzFRFLFLTLPRPRFLFLOROzFLPLFLTLPzCRCRFLTROROzPRFRCLFLCLORPRTRPRTRPLPLTRPLPLPRPLOLFLTROLFLFRTLCLOzCLOzPLOLFROzPLORFLOLTLFLFRFLFLCzPzOzCzFLTRFzTRFLPLCzOLFRTRPLPzTRFzFRFRFLPLFRCzPRFRPLPLFRFRFRFROROzPLTLPRPRCzFLFRORPzFRPRFRPRCRFLFLOzOLFLFzFLPzPRTLCRFRTRCLPzTRTRFzFRPzFRFLTRFRFLCRFRFLOzFLFLFRFRPRPzOzFLFROLFROzORORTRCROLFRFLFLTRCLPLPzPLOLOzPLPLPRPROLPRORPLPRPLCLFLORFRFRFRPLPRFRFRTLCRPLPRCLFRPLOzFRFRPLCLOzPzCzFRFRFRPRTLPzOzFROLOLPLPRTRCRFzFRTRFLPzPRPzPzCzFLTLTLPLFLFRPLORFRFROzTLORPRORPLTRORCLPLPLPzFROzFRFRPLOLFRFLFzPRFRFROzPLORPLCLFLPRPRFRPLPLCLCzFRPLCzPzPLOLPLFLFLFLFzTRFRCLPRTLPROzTRTLPRFRCzFLORTRFROLTLCRCzFLFLFRFLFRFRFLFLCLTLFRFzPzFLOzCRPRCRFRFzPLFRPzFLPRORPRCLFLCRCROLPLORORPRPLORPzPRFLTRCLCRFLCRFzFLCRPRTRORFRFRFLTRPROLTRFRFLFROzPRPRTLPzCLFLFLPzPRFLFzFLTLCROLFRTRCLPLPRORPLOzCRFLPLPLCRFRCRPRPLPRCROzFLCRFLFLCRFRPLPRFLPzOzCRPRORPzPLFRCROLPzFROLTLFLCzFRCLPLTLCLCRCLCLFLFLPzOzFLFLFROLCLFLFLFRFROLFRPROLFLPRPzOLCRFLCRCLFLTRPLFLTLPLPRFLPzPRPLPLOLFzPRPzCRFRPLCLPLPRPRORCzFLCLCzPROLFLFLFRPRCRPLPLPRCRFRPRPzPLPRFLFRFLFRCzORPRFRFLFRCROLORFLCLTLFLFRFLCLCLPzOzPLFLFLFRFRFRFLORFR.

Hemispheric sentence:

RzLRzLLLLLLLRRLLLzRRzRRRLLzRLLRRRRzRRRLzRRzLRRLLLLRLRRRLRRRRRRLLRLLRRLRRRzLLLzzzRRRLzLRRRzRLLRLLRRLLRRzLzRLRzLRRLLLRRzLLLRLLLLRRRLRLRLRRzRRRLLLzRRzLzRLLRLLzLLLLRLRLRRRLRLLLLLRRzRLRRzLzRRRRRRzLRLLLRRLzRRLLLLRzRzRLLLLRLRRLLLRLRLRLLLLLLLRLzLRLRRLzRRLRLLRRLzLRLzLRLLLzLLRRRzLRRRRRRLzLLLzLLLLLRRzLLRLRRzRLRLRRLLLLLLRRRRLRLLRzRRRLLLRRRLLRLzRRRLLzLzRLLLRRLRLzRRLLRRRRRRLRLRLLzLLLRRRRRRRRLLLLRRLRLLLRzRRLRRLLLLzzLRRzRzRLRRzRLRRzLzLLRRRzLzLRRRLzLRRRLLRLzRLLzLRLLLzRLzRLRRRLLRRzLLLRzRzRRzLLzLRzLzzzRRRLRRRzRLRRzRRRzLLLLLLRLzRLzLzRzRRzRLRLRRRRLRLLLRRRLRRRLLRRLLRRRRLzLLRRLLLLLRRLLRLLzRzzLLRLLRRRLLRzRRLLRRLRRRRzLLzRRRLzLRRRRRRRRRRRLLRRLLLLLRRRLLzRLRRLRRRRRzRLzRzLzLLzLLzLzLLRRRRRRRLRLLRzzzRLLLRRLLRzLLLLzRRLRRzRRLLLRRRRRLLRLLRLLLRLLRLLzLzLLRzLRLLLLRLLzzzzLRzRLLzLRRLzRzRRLLRzRRLLRRRRRzLLRRzLRRzRRRRRLLzLLzLzRLRRRLzRRzRzRLRRLRRLzLLRRRzzLRLRzRRRRLRLLRLLzLLzLLRRLRRLRLLLRRRRLRRRLRLRLRLzRRLLzzzRRRRLzzRLLLRRRzRRLzRzzzLLLLLRLRRRzLRRRLRRLLLzRzRRLLRLzRRRzLRLLLRRRLLLzRLzzLLLLLLzRRLRLRzRLRRzLRRRLLRzLLRLRRLLLLRzzLzRRRRzLRzLRRRLLRRLLRRRLRzRLRLRLRzLRRRRRRLRRLRRLRzRRLzLLLzRLzLLRLRRLLRRLzRLLLRRRRLRRzLRLLRRLRLzzRRRzLRRLzRLLLzRLLLLRLLLLzzLLRLLLLRRLRRLLRzLRLRLLRLLLLRLzRLLLzRzRRLLLRRRzLLzRLLLRRRLLRRRRzLRLRLRzRRRLRRLRLLLLRLLLzzLLLRRRLRR.

**Figure 7:**
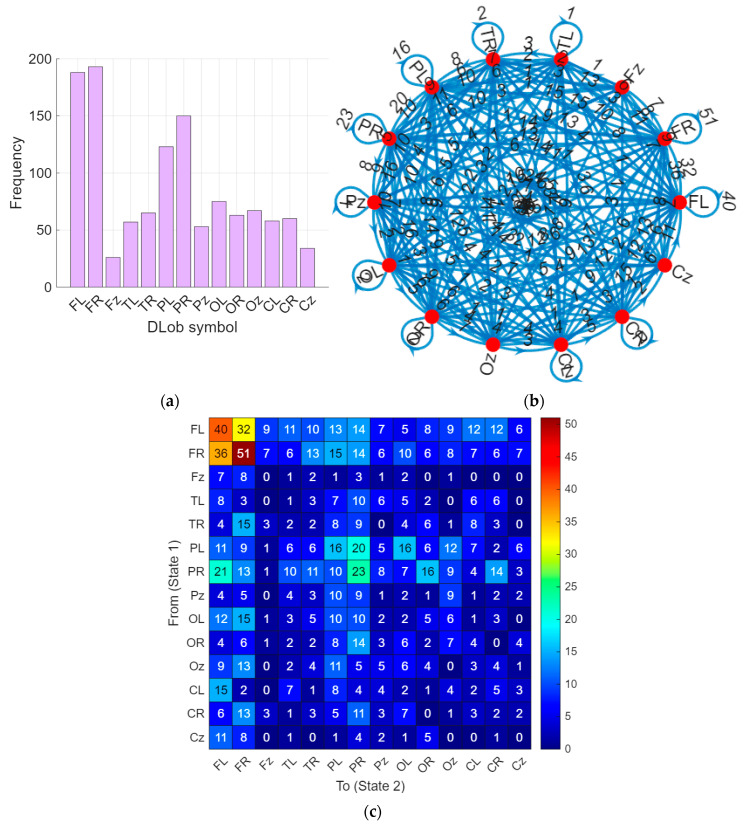

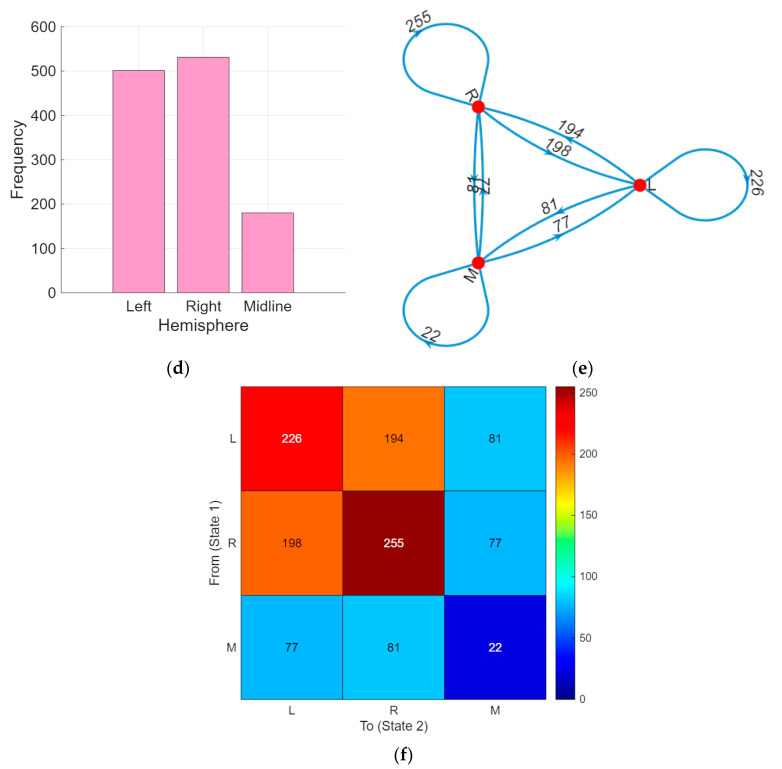
The graphical demonstration of the XAI results. (**a**) Histogram of the DLob sentence; (**b**) connectome diagram of the DLob sentence; (**c**) transition table of the DLob sentence; (**d**) histogram of the hemispheric sentence; (**e**) connectome diagram of the hemispheric sentence; (**f**) transition table of the hemispheric sentence.

Deploying these sentences, we have generated XAI results from these sentences, and the computed XAI results have been schematized in Figure 7.

**Figure 8:**
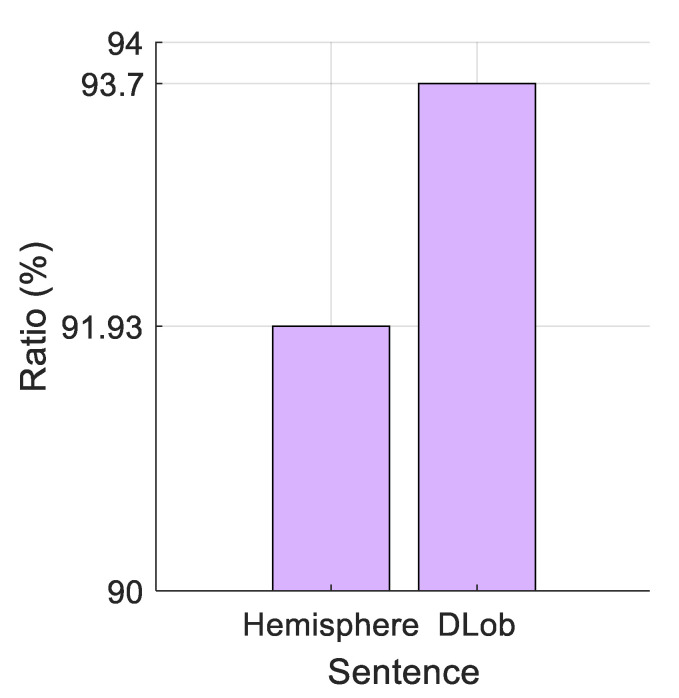
Complexity ratios of the generated sentences.

The complexity ratios of these sentences are demonstrated in Figure 8.

The complexity ratios clearly indicate that EEG-based odor processing is a complex process for the brain.

### 3.3. Results Obtained Using the Olfactory EEG Dataset

To evaluate generalization performance, experiments were conducted on the publicly available EegDot olfactory EEG odor-type classification dataset established by Tianjin University [36]. This dataset was selected because of its open accessibility and standardized acquisition protocol. The EegDot dataset contains thirteen odor classes: rose, caramel, rotten, canned peach, excrement, mint, tea tree, coffee, rosemary, jasmine, lemon, vanilla, and lavender.

**Figure 9:**
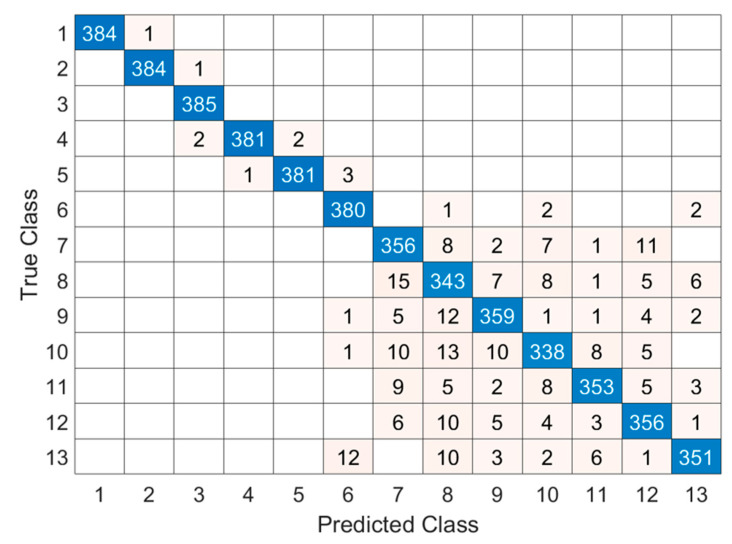
Confusion matrix for olfactory EEG datasets.

The classification performance obtained on the publicly available EegDot dataset is illustrated in Figure 9, where the confusion matrix demonstrates a strong diagonal dominance across all thirteen odor classes, indicating consistent class-wise discrimination.

As reported in Table 4, the proposed TensorCSBP XFE framework achieved an overall accuracy of 94.93%, with balanced sensitivity (94.93%) and high specificity (99.58%). The F1-score of 94.87% and geometric mean of 97.24% further confirm that the model maintains stable decision boundaries across multiple odor categories. These findings validate the generalization capability of TensorCSBP beyond the self-collected dataset and demonstrate its robustness in multi-class olfactory EEG classification scenarios.

## 4. Discussions

In this research, we collected a new EEG odor classification dataset and proposed an innovative feature-extraction method termed TensorCSBP. To investigate the feature-extraction ability of TensorCSBP, an XFE framework was recommended. In this framework, features were extracted using the TensorCSBP method. The most salient features were chosen with the CWNCA feature selector; tkNN generated the classification results, and DLob produced the XAI results.

**Figure 10:**
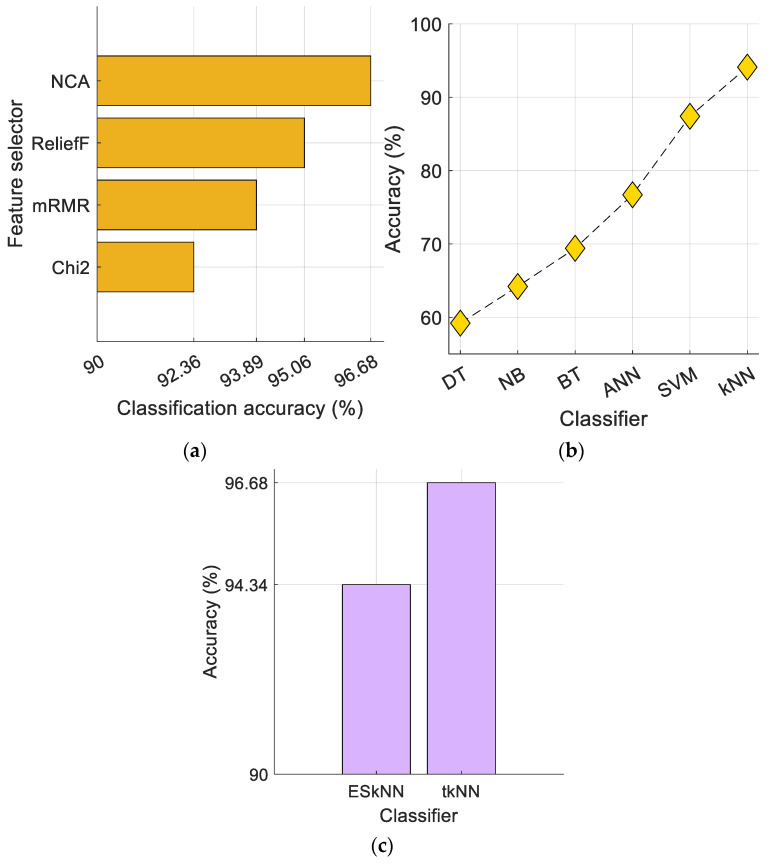
Comparative results. (**a**) Performance of the feature selectors; (**b**) performance of the shallow classifiers. Herein, DT: Decision Tree, NB: Naïve Bayes, BT: Bagged Tree, ANN: Artificial Neural Network, SVM: Support Vector Machine, kNN: k-nearest neighbors; (**c**) classification results of the Ensemble Subspace kNN (ESkNN) and tkNN.

To achieve high classification performance, TensorCSBP, CWNCA, and tkNN were used together. The tkNN classifier is a self-organized ensemble classifier. It generates 238 outcomes (120 parametric + 118 voted) and automatically selects the best one. To obtain the best classification results, we tested classifiers and feature selectors. The computed test results are shown in Figure 10.

Figure 10 clearly shows that the best feature selector is NCA and the best shallow classifier is kNN, as kNN reached 94.07% classification accuracy on the collected dataset. Therefore, we applied the t algorithm to the kNN classifier, and the tkNN classifier reached 96.68% accuracy. Moreover, we compared tkNN with ESkNN; tkNN achieved 2.34% points higher accuracy than the ESkNN classifier. To further benchmark the proposed framework, three representative methods from the literature (Table 1) were implemented on the collected dataset under the same 10-fold cross-validation protocol. The comparative results are presented in Table 5.

As shown in Table 5, the proposed TensorCSBP XFE framework outperformed all three baseline methods, confirming its effectiveness on the collected EEG odor dataset.

### 4.1. Test of Additional Datasets

The collected dataset is a new-generation dataset; therefore, there are no classification results from previous models. To create a comparative results table, we applied the recommended model to other EEG datasets. The datasets used are:

- EEG artifact classification dataset [17];
- EEG hunger detection dataset [37];
- Turkish EEG mental performance detection dataset [38];
- STEW dataset [39].

To compute the classification results for these datasets, we applied the presented TensorCSBP XFE model.

In the EEG artifact dataset, there are eight classes. The first class contains clean EEG signals, and the remaining seven classes are EEG signals with artifacts. In this dataset, there are seven artifact classes. This dataset was collected using a 14-channel brain cap; therefore, the introduced TensorCSBP extracts 784 features.

In the EEG hunger detection dataset, there are two classes: (1) hunger and (2) control. A 14-channel brain cap was used for this dataset. Thus, 784 features were extracted by the TensorCSBP feature extractor.

The third additional dataset is termed the Turkish Mental Performance Detection (TMPD) dataset, and it has two classes: (1) high mental performance and (0) low mental performance. The TMPD dataset was collected using a 32-channel brain cap. Therefore, the recommended TensorCSBP extracts 4096 features from each EEG signal in this dataset.

The fourth additional EEG signal classification dataset is the STEW dataset, a mental performance detection dataset like TMPD. It has two classes: (1) high performance and (0) low performance. The recommended TensorCSBP extracts 784 features, since this dataset was collected using a 14-channel brain cap.

**Figure 11:**
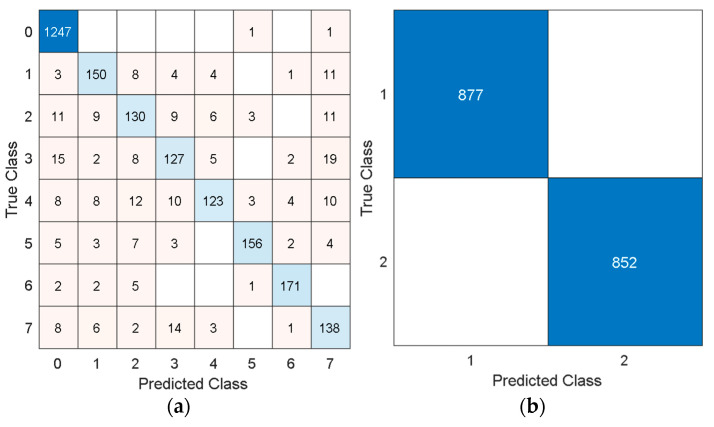

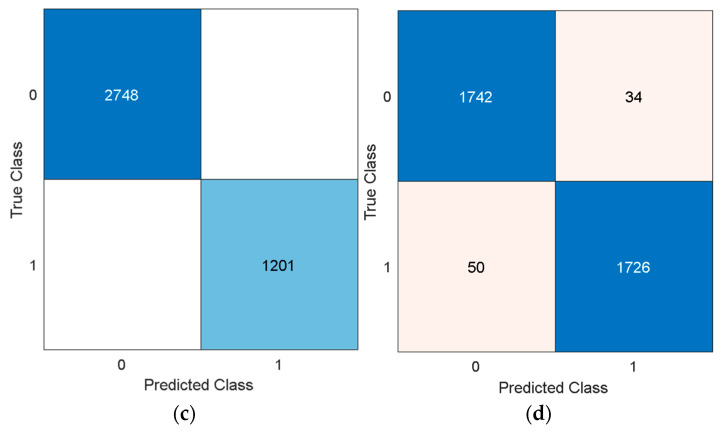
Confusion matrices of the four additional datasets utilized by deploying the TensorCSBP XFE model. (**a**) EEG Artifact Classification Dataset. Accuracy: 89.75%; (**b**) EEG hunger detection Dataset. Accuracy: 100%. (**c**) TMPD dataset. Accuracy: 100%; (**d**) STEW dataset. Accuracy: 97.64%.

The confusion matrices have been depicted in Figure 11.

We used these datasets to compare the classification performance of the TensorCSBP XFE framework with other EEG signal classification models, and the comparative results are given in Table 6.

Table 6 openly demonstrated that the recommended TensorCSBP XFE model attained high classification performance on the additional EEG datasets.

### 4.2. XAI Results Discussions

This study examines how the human brain represents smell and how explainable artificial intelligence can help us understand it. We used EEG recordings and turned them into simple symbols through a method called Directed Lobish, or DLob. A model named TensorCSBP XFE then sorted the smells and showed how the brain guided each decision.

When we examine the neuroanatomy of the sense of smell, we see that many different anatomical areas function together. The primary olfactory cortex is located in the uncus, in the medial temporal area. Structures comprising the primary olfactory cortex include the anterior olfactory nucleus, the olfactory tubercle, a small anteromedial portion of the entorhinal cortex, the periamygdaloid cortex, and various areas within the amygdala, such as the prefrontal cortical nucleus and the lateral olfactory tract nucleus. After anatomical and physiological evidence proved that structures in the primary olfactory center project to a number of secondary structures, including the caudal orbitofrontal cortex (OFC), the agranular insula, and the hippocampus, but also the dorsomedial nucleus of the thalamus, the medial and lateral hypothalamus, and the ventral striatum and pallidum, these structures were named the secondary olfactory cortex [49]. The primary olfactory cortex is represented in the EEG by the central, parietal, and frontocentral channels according to the 10–20 mounting system. The temporal channels in the EEG mainly represent the lateral temporal regions, and these areas primarily record information related to the comprehension and hearing centers. Central channels, due to their anatomical proximity, are involved in the registration of both primary and secondary olfactory cortex signals. Frontal channels largely represent the orbitofrontal cortex, which is located within the secondary olfactory cortex. The occipital channels represent occipital-associated areas of the olfactory cortex, such as the cuneus [50].

The piriform cortex comprises the largest area of the primary olfactory cortex. In humans, it is located in the paleocortex at the medial junction of the frontal and temporal lobes. It receives inputs from the olfactory bulb, anterior olfactory nucleus, and olfactory tubercle [51]. Various studies have shown that piriform cortex activation is associated with odor identity, intensity, quality, and categorization in healthy people [52,53,54]. There are also studies showing that imagining smells without any stimulus activates the frontal portion of the piriform cortex [55].

There is evidence that the amygdala, located in the limbic system in the medial temporal lobe, is active during both pleasant and unpleasant odors and is involved in the emotional processing of odors [52].

The medial prefrontal cortex, located in the secondary olfactory cortex, is an area that evaluates the social aspects of senses, while the orbitofrontal cortex is more involved in higher-level olfactory processing processes, such as the continuous evaluation of the sensory value of an odor, and in distinguishing different odors. The insula, on the other hand, shows reduced activity in the perception of unpleasant odors [52,56,57]. It plays a particularly important role in distinguishing between pleasant and unpleasant odors [58]. Furthermore, a study conducted on rodents showed that the activation of the orbitofrontal cortex increased in relation to the reward aspect of smell [59].

Paralimbic structures that are active during olfactory perception include the parahippocampal gyrus, entorhinal cortex, hippocampus, paracingulate gyrus, precentral gyrus, caudate nucleus, frontal pole, putamen, pallidum, and central opercular cortex. The activation of these brain regions, many of which support the emotional quality of olfactory stimuli, also demonstrates the existence of a network of structures involved in the processing of memory and emotion during olfaction [59].

DLob symbols mark activity in specific brain lobes and regions. The symbol frequency in each area shows which areas of the brain become most active when people perform smell-related tasks. The right and left frontal region symbols each activated close to 200 times (FL activated 188 times and FR activated 193 times). The research shows that the frontal cortex begins its operation when smell processing starts at its beginning stage [52]. Our findings are consistent with the literature, indicating that the right frontal region functions to detect dislike, but the left frontal region enables people to identify scents and perform intentional decisions [56].

The left and right parietal region symbols were seen 123 to 150 times. The brain processes smell as more than just a chemical signal, according to this information.

The left and right occipital region symbols appeared 63 to 75 times, mostly when the smell was unpleasant. Similar to our results, studies have shown through fMRI studies that the cuneus, located in the occipital cortex, is active in the early stages following olfactory stimulation and during the identification of the odor. The same study also found stimulation in the left-dominant bilateral frontal lobes and the right-dominant temporal, middle, and lower lobes during olfactory perception. The conclusion of this study, similar to ours, is that it begins with the development of a spatial representation of the stimulus, continues with the processing of more specific olfactory features, and concludes with a higher level of multisensory integration of the odor stimulus [52,60].

Symbols for the central brain areas were seen 34 to 60 times. The brain regions enable us to perform fast responses that lead us toward pleasant smells or away from unpleasant odors. The corpus callosum is recorded via central channels on EEG [50]. In an MRI study using rodents, it can be concluded that the smell of a desirable food stimulates the anatomical area called the tenia tecta, located ventral to the corpus callosum, more distinctly than the smell of an undesirable food, and this is consistent with the increased stimulation of central and parietal areas in response to pleasant smells [61].

We also looked at how the different brain areas talk to each other. The right frontal region showed 51 recurrent self-transitions, mostly during unpleasant smells. The left frontal region did this 40 times, mainly when naming or judging a smell [62]. The left and right parietal regions exchanged information 20 to 23 times. The central lobes shared signals 15 times.

Transitions between the left and right temporal regions were rare. The reason for these findings the temporal channels record signals from the lateral temporal area, where the auditory cortex and Wernicke’s area of cognition (the center for understanding) are located, rather than from the medial temporal area, where the primary olfactory cortex is located in EEG [50].

The right hemisphere showed 531 transitions, which mainly happened when people smelled unpleasant smells. This pattern may reflect avoidance-related processing and threat evaluation associated with unpleasant odors [56]. The left hemisphere contained 501 transitions that enable people to identify and state the names of different scents [56]. The midline region showed 180 transitions. Cross-talk between the two sides of the brain happened 194 and 198 times. The system shows how these two elements work together to create the complete sensory experience that perfumes provide to users.

We compared how much information is carried by DLob symbols that focus on single lobes and summaries that look at whole hemispheres. The DLob sentence had 93.7% complexity. The hemisphere summary had 91.93% complexity. The small difference shows that lobe-specific details add valuable information.

**Figure 12:**
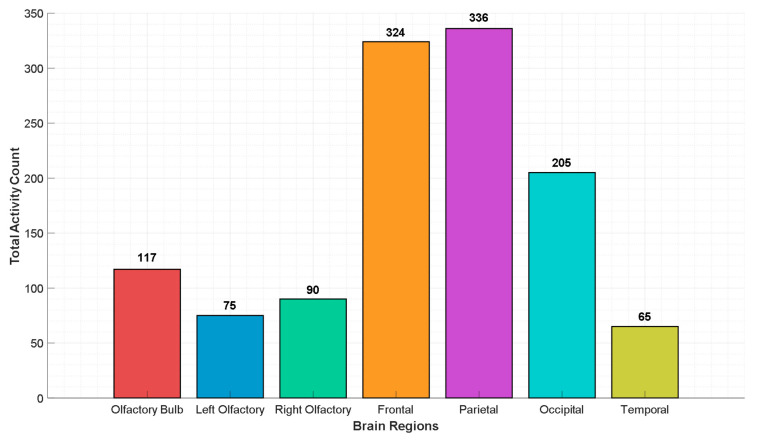
Total EEG activity across brain regions during olfactory processing.

By utilizing channel activation, we have presented olfactory network activation in Figure 12.

Figure 12 showcases the total activation levels in different brain regions during EEG-based smell/odor processing. The diagram also illustrates how the brain processes smell information, starting from the olfactory bulb, then moving to the right and left olfactory centers, and finally spreading toward the frontal and parietal regions.

The F3 and F4 electrodes represent frontal scalp locations that may reflect cortical activity associated with olfactory processing. F3 alone contributed 80, and F4 contributed 37. This region is the first area where the brain receives olfactory signals. The left and right centro-parietotemporal regions were then examined as scalp-level correlates of odor-related processing. The left center, made up of the T7 and C3 channels, had 75 total activations. The right center, including P8, P6, and C4, had a total of 90 activations. Together with the temporal channel T8, which had 65 activations, the overall odor-related activity reached 347 (=117 + 75 + 90 + 65). This highlights that the early olfactory centers were more active than other areas, indicating a strong initial brain response to smells before higher-level cognitive processing begins.

The frontal region, except for the primary olfactory channels F3 and F4, showed 324 total activity points. The frontal lobe seems to function as a critical area that determines how people process and understand different scents. The parietal region showed the highest activation rate because it contained 336 activations across channels P3 and P4 and all other channels except those in the right and left olfactory centers. The parietal lobe in the brain serves as a connection point that enables the processing of olfactory information together with other sensory inputs. Furthermore, this high stimulation number is due to the fact that, with 10–20 system EEG monitoring, primary olfactory cortex signals are recorded from the parietal channels along with the central electrodes rather than the temporal channels [50].

The occipital region, which contains channels O1, Oz, and O2, showed 205 activation events. This may indicate involvement of the visual processing areas in how people mentally respond to smells [52,60]. The T8 region showed low activation because it received 65 signals. The T8 area is anatomically congruent with the right auditory center [50].

The signal path followed this sequence from the olfactory bulb (117 activations) to the right and left centers (165 activations, which included 75 and 90 activations) before reaching the frontal (324) and parietal (336) channels associated with the primary olfactory cortex.

The right-to-left bulb ratio was calculated by dividing the total activation in the right and left olfactory centers by the activation in the olfactory bulb. The olfactory bulb had 117 activations in total. The right and left centers included the left side (T7 and C3) with 75 activations and the right side (P8, P6, and C4) with 90 activations. The total for the right and left centers was therefore 75 + 90 = 165. The ratio was computed as 165 ÷ 117 ≈ 1.41. The right and left centers operated at 41% higher activity levels than the bulb did.

The frontal-to-right and left ratio was calculated by comparing the activation in the frontal region (excluding F3 and F4) to the total in the right and left centers. The frontal region showed 324 activations, but the right and left centers together had 165 activations. The ratio was computed as 324 ÷ 165 ≈ 1.96. This shows that the frontal lobe was almost twice as active as the right and left olfactory centers during smell processing.

The parietal-to-frontal ratio was found by dividing the activation in the parietal region by that in the frontal region. The parietal region had 336 activations, and the frontal region had 324. The ratio between 336 and 324 equaled 1.03 when we performed the division. The study results indicated that both regions showed equivalent activity, while the parietal channels demonstrated slightly greater involvement.

The researchers determined the occipital-to-parietal ratio through a calculation that divided the number of occipital activation points by the number of parietal activation points. The occipital region (O1, Oz, O2) activated 205 times, but the parietal region activated 336 times. The result was 205 ÷ 336 ≈ 0.61. The occipital channels showed moderate involvement, less so than the parietal channels.

The olfactory network (which includes the bulb, right and left centers, and the temporal region) had a combined activation count of 347. This number was higher than the total counts from the frontal or parietal areas alone. The F3 channel, part of the left olfactory bulb, was the most active single channel in the study. The left side of the brain seems to handle smell processing more effectively, according to this finding. The frontal and parietal regions showed high activity, which supported their functions in processing smell-related information for interpretation.

Odors are not only detected in this research. They are also judged as pleasant or unpleasant. The researchers studied brain activity patterns between these two groups. The two types of stimulation activated the frontal region, but unpleasant odors produced greater right frontal brain activity. Unpleasant smells also triggered more body-related responses in the parietal channels associated with the primary olfactory cortex. A comprehensive meta-analysis revealed differences in the areas stimulated according to odor categories. For pleasant odors, high activity was recorded in the bilateral amygdala, right insula, left pallidum, left putamen, and central opercular cortex, extending to the piriform cortex, with the right hemisphere being more dominant, as well as the bilateral orbitofrontal cortex. In the analysis for repulsive odors, the highest probability of activation was found in the right hemisphere, with unilateral activations in the right hemisphere, particularly in the right insula, right postcentral gyrus, and right amygdala. In contrast, when food odors were analyzed, the highest probability of bilateral activation was found in the amygdala, extending to the piriform cortex. Unlike other odors, the activation cluster in this group was observed in the left hemisphere. Activation was also observed in the anterior part of the left parahippocampal gyrus and the left entorhinal cortex [56].

The temporal region displayed low activity levels, which maintained its ability to process memories and identify emotional connections [52,56].

In conclusion, all these stimulated anatomical areas indicate that the sense of smell is not simply a sensory experience but is subject to numerous higher cortical processes such as behavior, emotion, selectivity, categorization, and visualization, and that many associated areas, along with the primary and secondary olfactory centers, play a role in this processing.

### 4.3. Innovations and Contributions

Novelties:

- A new EEG odor detection dataset was collected.

The recommended TensorCSBP used a feature-extraction-dedicated transformer. To achieve this, we have presented a center-symmetric transformer (CSTrans). Four transformed signals were created from eight input vectors using CSTrans.

- By adding a transition table feature extractor (TTFE) to CSTrans, the TensorCSBP feature extractor was proposed. To our knowledge, this is the first center-symmetric feature extractor that generates features from a tensor.
- An innovative XFE (TensorCSBP XFE) model was presented to investigate the feature-extraction capability of the introduced TensorCSBP.

*Contributions:*

- The introduced TensorCSBP XFE architecture attained over 95% classification accuracy. Therefore, this XFE model contributes to feature engineering by achieving high accuracy on EEG odor detection.
- In the literature, most researchers have focused on increasing classification performance in EEG odor classification. However, there is a lack of XAI in EEG odor detection/classification. Moreover, there is no research on EEG odor detection using DLob [17] XAI. Herein, we used DLob to extract XAI results for odor. In this respect, this research contributes to neuroscience on odor detection.

### 4.4. Key Points

Findings:

- A two-class EEG odor dataset (good vs. bad) was collected: 1113 segments (571 good, 542 bad) from 180 participants aged 18–49 (16 female, 164 male). Signals were recorded with a 32-channel Emotiv Flex (256 Hz) and segmented into 15-s epochs.
- The TensorCSBP XFE pipeline achieved 96.68% accuracy (10-fold CV) on the odor dataset. Linear time complexity was confirmed, so all experiments were executed on a standard laptop (Intel i9, 32 GB RAM, MATLAB R2025a).
- Generalization ability was validated on four additional EEG datasets using the same pipeline, and the computed results demonstrated robustness across different tasks and hardware settings.
- The computed results indicated robustness across tasks (artifact removal, hunger state, cognitive performance) and hardware settings (14 vs. 32 channels).
- DLob-based XAI outputs were generated: the DLob symbol-sequence entropy was 3.5675; overall DLob sentence complexity reached 93.70%, while hemisphere-level summaries showed 91.93% complexity, indicating that lobe-specific detail added non-redundant information.
- Symbol frequencies highlighted frontal dominance: FR appeared 193 times and FL 188 times, revealing strong bilateral frontal engagement during odor processing (avoidance/valuation vs. naming/appraisal).
- Parietal and central symbols may reflect activity associated with networks involved in olfactory processing.
- Occipital symbols (OL/OR) appeared 63–75 times, predominantly for unpleasant odors, implying coupling between negative olfactory input and visual scanning/avoidance.
- Central symbols (CL/CR) appeared 34–60 times. These symbols, along with parietal symbols, indicate the localization of the primary olfactory cortex.
- Intra-lobe self-transitions were frequent: FR→FR occurred 51 times (mainly unpleasant odors), and FL→FL 40 times (labeling/judgment).
- Inter-lobe exchanges were moderate: PL↔PR occurred 20–23 times; central-lobe exchanges appeared 15 times. Temporal-lobe transitions were rare, consistent with a non-auditory/language task focus.
- Hemisphere-level transitions were asymmetric: right hemisphere 531, left hemisphere 501, and midline 180. Cross-hemispheric transfers occurred 194 and 198 times, evidencing bilateral cooperation.

Advantages:

- Explainability was ensured through DLob-based symbolic analysis at the lobe and hemisphere levels.
- A tensor-based, center-symmetric feature extractor (TensorCSBP) was introduced for the first time, enabling structured multi-channel processing.
- Feature dimensionality was efficiently reduced via CWNCA, improving robustness and reducing computation.
- A self-organizing classifier (tkNN) was employed to provide parameter exploration and majority-vote stability without manual tuning.

Limitations:

- Class and gender imbalance (16 females vs. 164 males) was present in the collected dataset.
- Only binary odor categories were considered, limiting the granularity of olfactory perception analysis.

Future works:

- Expansion to multi-class odor intensity and valence levels could be investigated.
- Cross-device and cross-laboratory validations could be conducted to assess robustness and reproducibility.
- Multimodal integration (e.g., fNIRS, EOG, respiration) could be explored to enrich odor-related neural signatures.
- Real-time deployment and on-device optimization could be pursued for BCI applications.

Potential implications:

- Consumer preference prediction in neuromarketing could be enabled by FL–PL EEG activity patterns.
- Depression-related olfactory sensitivity could be screened using right-frontal self-loops (FR→FR) as a biomarker.
- Understanding of odor processing pathways in neuroscience could be advanced through DLob-based symbol-level brain mapping.
- Integration of realistic olfactory feedback into VR/AR BCI interfaces could be guided by DLob symbols.
- Real-time detection of harmful odor leaks in industrial and laboratory settings could be supported by EEG-based monitoring systems.
- Objective assessment of olfactory function in clinical neurology and ENT practice could be standardized with the proposed framework.
- Regulatory compliance and user trust in biomedical AI could be strengthened through transparent, explainable analysis pipelines.

## 5. Conclusions

This study introduces the TensorCSBP feature extractor for EEG-based odor detection. An explainable feature-engineering framework (TensorCSBP XFE) was built around it. The framework used CWNCA for feature selection, tkNN for classification, and DLob for symbolic explanations.

A new two-class EEG odor dataset (good vs. bad) with 1113 segments was collected. TensorCSBP XFE reached 96.68% accuracy under 10-fold cross-validation. All steps had linear time complexity, so the pipeline ran on standard hardware.

DLob analysis produced clear and quantitative explanations. Frontal lobes dominated the decisions. Parietal and central midline regions may reflect activity associated with olfactory processing networks. Furthermore, parietal regions supported multimodal integration. The DLob sentence entropy (3.5675) and close complexity scores at lobe and hemisphere levels showed that detailed symbols added useful information.

The same pipeline was tested on four additional EEG datasets and maintained high accuracy. This result showed good generalization across different tasks and channel counts.

Two main limitations were noted: class/gender imbalance in the new dataset and the use of only two odor categories. Future work may include more odor types, balanced cohorts, and multimodal signals.

The introduced TensorCSBP XFE framework provided an accurate, efficient, and explainable solution for EEG odor detection. The approach can support clinical olfactory assessment and applications in neuromarketing, safety monitoring, and BCI/VR systems.

## Figures and Tables

**Table 1:** Olfactory EEG classification studies: method and accuracy comparison.

Study	Task	Model/Algorithm	Feature Extraction or Learning Strategy	Accuracy (%)
Hou et al., 2022 [22]	Pleasantness recognition from EEG elicited by five concentrations of pleasant (rose) and unpleasant (rotten) odors, plus binary pleasant vs. disgust	SVM	γ-band power-spectral-density features computed for each trial and fed to SVM (benchmarked against NB, kNN, ELM, BPNN)	93.5% (rose), 92.2% (rotten) for five-level tasks; 99.9% for binary pleasant vs. disgust
Kato et al., 2022 [23]	Ten-class identification of perceptually diverse odors	Time-resolved multivariate pattern analysis (ℓ2-regularized least-squares pairwise decoder and multinomial logistic regression)	64-channel OERP amplitudes concatenated in a 200 ms sliding window (50 ms step) to capture temporal dynamics	54.6% peak pairwise (chance 50), 13.6% peak ten-class (chance 10)
Pandharipande et al. (2023) [24]	Odor-independent EEG-based biometric identification	SVM	PyWavelets features	83.03%
Xia et al., 2023 [25]	Eight-class odor ID and binary pleasantness detection	SVM	Mutual-information functional brain network built from each trial; node-degree vector used as feature set	95.78% (8 classes), 98.21% (pleasant vs. unpleasant)
Naser & Aydemir, 2023 [26]	Imagined odor pleasant vs. unpleasant classification	kNN	Instantaneous amplitude derived via Hilbert transform after optimal band-pass filtering; random sub-sampling cross-validation for feature selection	87.75% mean across ten subjects
Aydemir, 2020 [27]	Four-class odor identification and participant identification (eyes-open/eyes-closed)	kNN	Band power, statistical, Hjorth, and autoregressive features; AR + kNN best for odor, statistical + kNN best for subject ID	96.94% (odor), 99.34% (subject)
Guo et al., 2024 [28]	Food-odor ID (eight classes) and pleasantness recognition	Ensemble (Random Forest + Extreme Learning Machine + PSO-optimized SVM)	34 EEG features ranked by ReliefF, mRMR, and ILFS, projected with K-PCA; sub-model outputs fused by voting	96.1% (odor), 98.8% (pleasantness)
Ouyang et al., 2025 [29]	Personal identification from multisensory (video + odor) emotion-evoked EEG (negative, positive, neutral)	Deep CNN followed by three-layer Bi-LSTM with residual links (CBR-Net)	CNN extracts spatial maps from 28-channel EEG; Bi-LSTM models temporal context before softmax classification	96.59% (negative), 95.42% (positive), 94.25% (neutral)
Fang et al., 2025 [30]	Low- vs. high-arousal recognition while inhaling sandalwood vs. bergamot essential oils	Random Forest (benchmarked against Discriminant Analysis and SVM)	Mean PSD from six sub-bands plus β/α arousal ratio across five regions of interest; partial least-squares used for dimensionality reduction before RF	95.0%

EEG: Electroencephalography; PSD: Power Spectral Density; SVM: Support Vector Machine; NB: Naïve Bayes; k-NN: k-Nearest Neighbour; ELM: Extreme Learning Machine; BPNN: Back-Propagation Neural Network; OERP: Olfactory Event-Related Potential; MVPA: Multivariate Pattern Analysis; L2-LS: L2-Regularized Least Squares; MLR: Multinomial Logistic Regression; AR: Autoregressive; RF: Random Forest; PSO: Particle Swarm Optimization; K-PCA: Kernel Principal Component Analysis; mRMR: Minimum Redundancy Maximum Relevance; ILFS: Infinite Latent Feature Selection; PLS: Partial Least Squares; CNN: Convolutional Neural Network; Bi-LSTM: Bidirectional Long Short-Term Memory; CBR-Net: Convolutional Bi-LSTM Residual Network; ROI: Region of Interest.

**Table 2:** DLob symbols.

No	Symbol	Area	No	Symbol	Area
1	FL	Left Frontal	9	PL	Left Parietal
2	FR	Right Frontal	10	PR	Right Parietal
3	Fz	Midline Frontal	11	Pz	Midline Parietal
4	TL	Left Temporal	12	OL	Left Occipital
5	TR	Right Temporal	13	OR	Right Occipital
6	CL	Left Central	14	Oz	Midline Occipital
7	CR	Right Central	15	AL	Left Auditory
8	Cz	Midline Central	16	AR	Right Auditory

**Table 3:** Classification performance evaluation metrics for collected dataset.

Classification Assessment Metrics	Result (%)
Accuracy	96.68
Sensitivity	97.90
Specificity	95.39
Precision	95.72
F1-score	96.80
Geometric mean	96.91

**Table 4:** Classification performance evaluation metrics.

Classification Assessment Metrics	Result (%)
Accuracy	94.93
Sensitivity	94.93
Specificity	99.58
Precision	95.61
F1-score	94.87
Geometric mean	97.24

**Table 5:** Comparison with representative literature methods on the collected EEG odor dataset (10-fold CV).

Study	Feature Extraction	Classifier	Accuracy (%)
Hou et al. [22]	γ-band PSD	SVM	88.77
Naser & Aydemir [26]	Hilbert amplitude	kNN	85.63
Guo et al. [28]	Statistical + ReliefF	RF + ELM + SVM	91.46
**This Paper**	**TensorCSBP + CWNCA**	**tkNN**	**96.68**

**Table 6:** Comparison of other EEG datasets with the TensorCSBP method.

Dataset	Study	Method	Validation	Result(s)	DL/ML	XAI
Artifact [17]	Tuncer et al., 2024 [17]	TTPat, CWINCA, tkNN, DLob	10-fold CV	Acc. = 77.58	ML	Yes
	Gelen et al., 2025 [40]	OTPat, CWINCA, tkNN, DLob	10-fold CV	Acc. = 86.07	ML	Yes
	**This Paper**	TensorCSBP, CWNCA, tkNN, DLob	10-fold CV	Acc. = 89.75	ML	Yes
Hunger [37]	Kirik et al., 2024 [37]	DSWIN, INCA and IRF, kNN, IHMV	10-fold CV LOSO CV	10-fold CV: Acc. = 99.54 LOSO CV: Acc. = 82.71	ML	No
	Tuncer et al., 2024 [17]	TTPat, CWINCA, tkNN, DLob	10-fold CV	Acc. = 98.55	ML	Yes
	**This Paper**	TensorCSBP, CWNCA, tkNN, DLob	10-fold CV LOSO CV	10-fold CV: Acc. = 100 LOSO CV: Acc. = 80.57	ML	Yes
TMPD [38]	Ince et al., 2025 [38]	CubicPat, CWINCA, tkNN, DLob	10-fold CV LOSO CV	10-fold CV: Acc. = 99.70 F1. = 99.79 Gm. = 99.62 LOSO CV: Acc. = 87.79 F1. = 91.58 Gm. = 82.0	ML	Yes
	**This Paper**	TensorCSBP, CWNCA, tkNN, DLob	10-fold CV LOSO CV	10-fold CV: Acc. = 100 LOSO CV: Acc. = 83.04	ML	Yes
STEW [39]	Safari et al., 2024 [41]	dDTF, RF, Forward FS, mRMR, SVM	7-fold CV	Acc. = 89.53	ML	No
	Jain et al., 2024 [42]	VMD, LightGBM	5-fold CV 10-fold CV	5-fold CV: Acc. = 95.51 10-fold CV: Acc. = 96.0	ML	No
	Yedukondalu and Sharma, 2023 [43]	Ci-SSA, BDA, kNN	10-fold CV	Acc. = 96.67 F1. = 96.90	ML	No
	Siddhad et al., 2024 [44]	ConvNeXt	Hold-out CV (70:15:15)	Acc. = 95.28	DL	No
	Parveen and Bhavsar, 2025 [45]	CNN Transformer, MV	5-fold CV	Acc. = 85.46	DL	No
	Yu and Chen, 2024 [46]	DMAEEG	5-fold CV	Acc. = 98.7	DL	No
	Yedukondalu et al., 2025 [47]	R-LMD, BAO, OEL	10-fold CV	Acc. = 96.1 Sen. = 96.0 Spe. = 97.0	ML	No
	Han et al., 2025 [48]	Functional connectivity features, PCA, LSTM	Hold-out CV (80:20)	Acc. = 96.64 Pre. = 97.21 Rec. = 96.53 F1. = 96.86	ML	Yes
	**This Paper**	TensorCSBP, CWNCA, tkNN, DLob	10-fold CV	10-fold CV: Acc. = 97.64	ML	Yes

Acc.: Accuracy, F1.: F1-score, Gm.: Geometric mean, Pre.: Precision, Rec.: Recall, Sen.: Sensitivity, Spe.: Specificity, ML: Machine Learning, DL: Deep Learning.

## Data Availability

The dataset can be downloaded at https://www.kaggle.com/datasets/buraktaci/odor-eeg (accessed on 23 February 2026).
